# Supplementary material for: Netcombin: An algorithm for constructing optimal phylogenetic network from rooted triplets
Source: PLoS One. 2020 Sep 18;15(9):e0227842. doi: 10.1371/journal.pone.0227842 (PMC7500971; doi:10.1371/journal.pone.0227842)
Supplement: S4 File — (ZIP) [file pone.0227842.s004.zip › SequenceData-Triplet/sequenceData-10-20-30-0-Triplet/length 200, numseq 40, rate 10^-7/5 seq.pdf]

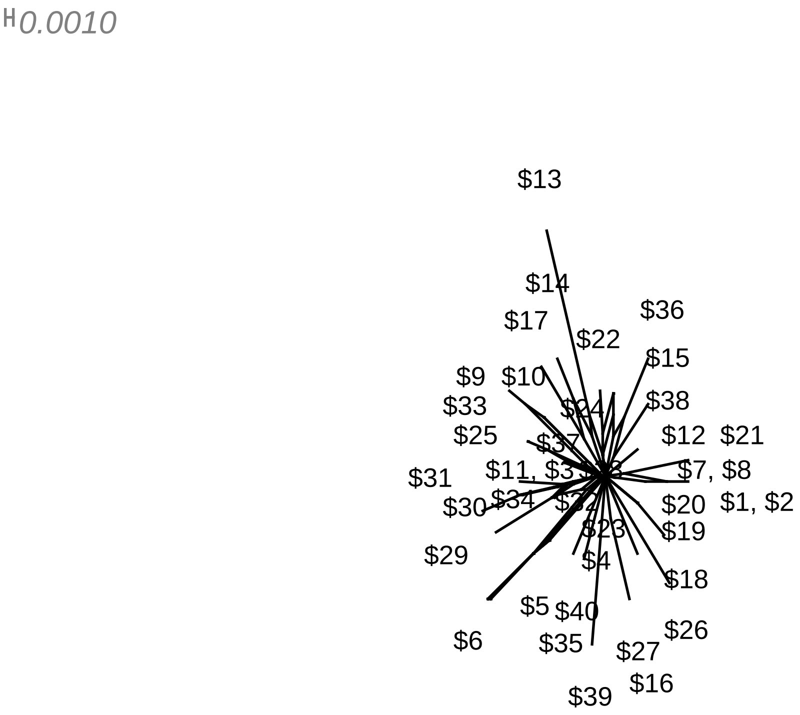
0.0010
$13
$14
$36
$17
$22
$15
$9
$10
$33
$24
$38
$25
$37
$12
$21
$11, $3
$7, $8
$31
$30
$34
$32
$20
$1, $2
$23
$19
$29
$4
$18
$5
$40
$26
$6
$35
$27
$16
$39
